# Determinant factors of pregnant mothers’ knowledge on mother to child transmission of HIV and its prevention in Gondar town, North West Ethiopia

**DOI:** 10.1186/1471-2393-12-73

**Published:** 2012-07-28

**Authors:** Marelign Tilahun Malaju, Getu Degu Alene

**Affiliations:** 1 Department of Public Health, College of Medicine and Health Sciences, Arba-Minch University, P.O. Box: 21, Arba-Minch, Ethiopia; 2 School of Public Health, College of Medicine and Health Sciences, University of Gondar, P.O. Box: 196, Gondar, Ethiopia

## Abstract

**Background:**

Mother-to-child transmission of HIV is a very important mode of HIV transmission for children. Well-functioning and accessible health facility and knowledge on mother to child transmission of HIV are a prerequisite for a successful mother to child transmission prevention of HIV. However, the determinant factors of pregnant mothers’ knowledge towards mother to child transmission of HIV and its prevention is not well studied in Ethiopia and particularly in the present study area.

**Methods:**

Cross-sectional health institution based study was conducted in Gondar town from July 22-August 18, 2011. A total of 400 pregnant women attending antenatal care (ANC) were involved in the study using stratified sampling technique. Data were collected by using structured questionnaire and multiple logistic regression analysis was used.

**Results:**

A total of 400 pregnant women actively participated in this study and 354 (88.5%) of them knew mother to child transmission of HIV and 334(83.5%) of them knew mother to child transmission of HIV is preventable. Having knowledge on mother to child transmission of HIV was positively associated with attending antenatal care visits in hospitals [Adj. OR (95%CI) = 4.49 (1.003, 20.06)], residing in urban areas [Adj. OR (95%CI) = 2.46 (1.19, 5.09)] and having education level of secondary and above [Adj. OR (95%CI) = 6.85 (1.96, 24.01)], but negatively associated with increased maternal age. Knowledge on prevention of mother to child transmission of HIV was positively associated with accessibility of health facility [Adj. OR (95%CI) = 2.16 (1.03, 4.57)], having perceived risk of HIV [Adj. OR (95%CI) = 2.61 (1.32, 5.17)], having comprehensive knowledge on HIV [Adj. OR (95%CI) = 2.86 (1.41, 5.82)], having education level of secondary and above [Adj. OR (95%CI) = 6.15 (1.75, 21.66)] and residing in urban areas [Adj. OR (95%CI) = 3.62 (1.73, 7.59)] but negatively associated with increased maternal age.

**Conclusion:**

Most of the study participants in this study knew that HIV could be transmitted from an infected mother to her baby. There should be well functioning and accessible health facilities with Prevention of mother to child transmission service in the country especially in the rural areas.

## Background

HIV still remains a major challenge globally despite decades of advocacy, awareness raising and investing in programs to control the spread of HIV. UNAIDS estimated that by 2009, 33.3 million people globally were living with HIV of which 22.5 million were from sub-Saharan Africa. UNAIDS also estimated that within the same period, about 2.5 children globally were living with HIV of which 1.8 million were from sub-Saharan Africa [1].

In sub-Saharan Africa an estimated 60% of people living with HIV are women, mostly in the reproductive age group. Each year approximately 1.4 million women living with HIV become pregnant. Among antenatal clients in sub-Saharan Africa, the proportion of women living with HIV ranges from 5% to as high as 30% and HIV among childbearing women is the main cause of infection among children [2].

In 2009 the national HIV prevalence was estimated at 2.3% with differentials: urban (7.7%), rural (0.9%), male (1.8%), and female (2.8%). The number of people living with HIV/AIDS was 1,116,216 of which 84,189 were pregnant women, 72,945 were children under 15 years and annual HIV positive births were 14,140 [3].

The risk of transmission varies at different stages ranging from 5% - 10% during pregnancy, 10% - 20% during labor and delivery, and 10% - 20% through mixed infant feeding. It is estimated that in the absence of any intervention to prevent mother to child transmission (MTCT) ranges from 15% - 45%. This rate can be reduced to levels below 5% with effective interventions [4].

The World Health Organization (WHO) promotes a three pronged approach to reduce mother to child transmission (MTCT) of HIV. Therefore: the prevention of new infections in parents, avoiding unwanted pregnancies in HIV infected women (primary preventions) and preventing transmission of HIV from an infected mother to her infant (secondary preventions) [5].

Nowadays, with a combination of anti-retroviral prophylaxis, elective caesarean section and by abstinence from breastfeeding it is possible to reduce mother to child transmission (MTCT) of HIV to <2% in developed countries. As this is not still possible in resource-poor countries, primary prevention is considered as the most important way to decrease mother to child transmission (MTCT) of HIV [6]. An increasing incidence of HIV in pregnant mothers would ultimately lead to increased incidence of HIV in children. The reasons for an increasing mother to child transmission (MTCT) of HIV might include lack of knowledge of mothers on the risk of mother to child transmission (MTCT), lack of access to provider health initiative and the benefits of preventive interventions, like antiretroviral (ARV) drugs and infant feeding options. Mothers should be aware about HIV/AIDS and its routes of transmission to children and they should be motivated to recognize their sero-status and be advised on primary and secondary preventive measures. However, the awareness of mothers on mother to child transmission of HIV and its prevention is not well studied in Ethiopia and particularly in the present study area. Therefore, this study was conducted to identify the determinant factors of mothers’ knowledge on mother to child transmission of HIV and its prevention.

## Methods

Health institution based cross-sectional quantitative study was conducted from July 22 – August 18, 2011. The study was conducted in Gondar town which is located about 750 km northwest from Addis Ababa the capital city of Ethiopia. The town has 12 administrations and a population of 220,184 and of this 51,963 was females of reproductive age group (15–49 years) with in the area of 41.27 square Km. There is one referral hospital and five health centers which offer antenatal care (ANC) and prevention of mother to child transmission of HIV (PMTCT) services in the town. The source population was all pregnant women attending antenatal care in public health facilities since these health facilities serve the majority of the population in antenatal care (ANC) service especially the rural and poor population. The study population was all pregnant women attending antenatal care during the data collection period in public health facilities of Gondar town, Northwest Ethiopia.

### Sampling procedure

Five health centers and one hospital found in Gondar town were included in the study. Stratified sampling technique was used to select the study units in each health institution. Based on the number of customers who visited each health institution during the previous ten months (monthly report of each health institution), proportional allocation of the total sample size was carried out to attain the required sample size in each health institution. Finally, the determined sample for each health institution was achieved through exit interview from systematically sampled and voluntarily consenting pregnant women with in four weeks of working days. Pregnant women attending antenatal care in health institutions of Gondar town, Northwest Ethiopia during the data collection period was included in the study.

### Data collection and quality control

Clinic staffs providing antenatal care (ANC) services were trained on the data collection and interview techniques. Interviewer administered structured questionnaire was used to collect the data. The designed questionnaire was translated first into the local/national language (Amharic) and back translated to English to ensure its consistency. The questionnaires were pretested in similar settings two weeks before the data collection and after finalized modification it was used to elicit the following information from the study participants: socio-demographic data, knowledge on mother to child transmission (MTCT) and prevention of mother to child transmission (PMTCT) of HIV, number of antenatal care visits, comprehensive knowledge on HIV/AIDS, risk perception of HIV, perceived benefit of HIV test and stigmatizing attitude towards people having HIV/AIDS. The completeness and consistency of data was established through direct and daily supervision by the supervisor and principal investigator. Data coding, cleaning and verification were performed by cross-checking the printout data for obvious errors, (i.e. exceptionally long or short lines, blanks that should not be there, alphabetic codes where numbers are expected, obviously wrong codes) to assure quality of data.

### Data processing and analysis

Sample size was determined using the formula of a single population proportion estimation and calculated using software Epi-info stat calc. by taking 59% proportion, 5% of absolute precision and with 95% confidence interval. Non-response rate in this study was estimated to be 10% i.e. 38, and hence an overall sample size of 410 Pregnant women were recruited in the study. Data were entered and analyzed using SPSS software version 16. Descriptive statistics such as frequencies and proportion was used to describe the study population in relation to relevant variables. Explanatory variables found to be statistically significant in bivariate logistic regression analysis were entered into multiple logistic regression analysis (backward stepwise method) for adjustment of confounding effect between explanatory variables (educational status, age, residence, number of antenatal care visits, comprehensive knowledge on HIV/AIDS, risk perception of HIV, perceived benefit of HIV test, accessibility of health services and stigmatizing attitude towards people having HIV/AIDS). Odds ratio, confidence interval and P-value were computed to assess the presence and degree of association between dependent variables (knowledge on mother to child transmission (MTCT) and prevention of mother to child transmission (PMTCT) of HIV) and independent variables (age, residence, education level, type of health facility, perceived risk of HIV, comprehensive knowledge on HIV and accessibility of health facility).

### Ethical consideration

Ethical clearance to conduct the study was obtained from Ethical Review Board, School of public health, University of Gondar and permission to conduct the study in each health facilities was secured from the respective Health institutions in Gondar Town. Verbal informed consent from each study participants was obtained after clear explanation about the purpose of the study.

## Results

From the total 410 pregnant women recruited 400 participated actively in this study in health facilities of Gondar town making the response rate of 97.6%. About 285 (71.2%) of the study population were from urban areas while the rest 115 (28.8%) were from rural part of the study area.

Over 90% (361) of the respondents were married followed by those who were divorced/separated/widowed 20(5%) and the majority 194(48.5%) of them had no education, followed by those who attended school secondary and above education 168(42%). The most frequent occupation was housewife (59%) seconded by government employed (16%) and merchant (10.2%), respectively.

The majority 391 (97.8%) of the study participants were Amhara by ethnicity followed by Tigre and Gurage. Most of the study participants 361 (90.2%) were followers of orthodox Christianity followed by Muslim 35(8.8%).

Regarding their age distribution 184(45.8%) of them were in the age range between 25- 34 years with mean (±SD) age of 25.37(±5.25) and majority (48%) of them have monthly expenditure of 451-999 birr per month (Table 1).

**Table 1:** Socio-demographic Characteristics of pregnant women attending ANC in Health Facilities of Gondar Town, North west Ethiopia, 2011

**Variables**	**Number**	**Percent**
**Age **[Mean(±SD) = 25.37(±5.25)]		
15-24	178	44.5
25-34	184	46.0
35-49	43	10.8
**Residence**		
Urban	285	71.2
Rural	115	28.8
**Ethnic group**		
Amhara	391	97.8
Tigray	8	2.0
Gurage	1	0.2
**Religion**		
Orthodox Christian	361	90.2
Muslim	35	8.8
Protestant	4	1.0
**Education**		
No education	194	48.5
Primary Education	38	9.5
Secondary & above	168	42.0
**Occupation**		
Government employed	64	16.0
Merchant	41	10.2
House wife	236	59.0
Student	37	9.2
Others*	22	5.5
**Marital Status**		
Never married	19	4.8
Married/living together	361	90.2
Divorced/separated/ widowed	20	5.0
**Monthly Expenditure**		
≤450 birr/month	107	26.8
451-999 birr/month	192	48.0
≥1000 birr/month	101	25.2

* Bartender, daily laborer and Jobless.

### Knowledge of pregnant women on mother to child transmission of HIV and its prevention

Regarding knowledge of mother to child transmission and prevention of HIV/AIDS, most of them 354(88.5%) knew that HIV can be transmitted from a mother to her child and 334 (83.5%) of them knew that mother to child transmission (MTCT) of HIV is preventable. As to the period of HIV transmission, 35.9% of mothers said it occurs during pregnancy, 33.6% of mothers responded that it occurs during labor and 24.9% of respondents said that it occurs while breast feeding. On the other hand 58.4% of mothers knew the protective effect of antiretroviral drugs, 18% of participants knew that abstinence of breast feeding can prevent mother to child transmission (MTCT) of HIV and 11% of mothers knew that elective cesarean section delivery can prevent MTCT of HIV (Table 2).

**Table 2:** Knowledge of respondents on MTCT & PMTCT of HIV among pregnant Women attending ANC in Health facilities of Gondar Town, North west Ethiopia, 2011

**Variables**	**n = 400**
**Those women who:**	**No (%)**
**know MTCT**	354 (88.5)
**know PMTCT**	334 (83.5)
**say that MTCT could occur:**	**n =354**
During pregnancy	127 (35.9)
During childbirth	119 (33.6)
Through breast feeding	88 (24.9)
Multiple answers	30 (8.5)
**say that PMTCT is possible by:**	**n =334**
Use of anti-retroviral drugs	195 (58.4)
Avoiding breast feeding	60 (18.0)
Safe delivery	37 (11.0)
Multiple answers	61 (18.3)

### Determinant factors of pregnant mothers’ Knowledge on mother to child transmission of HIV and its Prevention

In order to measure the association of pregnant mothers’ Knowledge on MTCT and PMTCT of HIV with a number of explanatory variables, crude OR and adjusted OR with 95% CI were employed. After controlling for confounders, the association between selected explanatory variables and Knowledge on MTCT and PMTCT of HIV is presented in Tables 3 and 4.

**Table 3:** Association between knowledge of pregnant mothers on MTCT of HIV and each explanatory variable (Crude & adjusted OR), Gondar, North West Ethiopia, 2011

**Explanatory Variable**	**Knowledge on MTCT of HIV**		**Crude OR (95% CI)**	**Adjusted OR (95% CI)**	**P-value**
	Yes(1)	No(0)			
**Age**					0.008
15-24	171	7	8.724(3.068, 24.814)	4.477(1.453, 13.791)	
25-34	155	29	1.909 (.838, 4.350)	1.142 (0.465, 2.808)	
35-49	28	10	1.00	1.00	
**Residence**					
Urban	269	16	5.934 (3.086,11.411)	2.459(1.189, 5.087)	0.015
Rural	85	30	1.00	1.00	
**Education level**					0.011
No Education	155	39	1.00	1.00	
Primary education	34	4	2.139(0.716, 6.386)	1.220(0.374, 3.977	
Secondary & above	165	3	13.839(4.191,45.698)	6.853(1.956, 24.014)	
**Type of health facility**					
Hospital	99	2	8.541(2.032, 35.903)	4.486(1.003, 20.061)	0.050
Health center	255	44	1.00	1.00	

➢ For explanatory variables having more than two categories, the overall significance is given by their corresponding P-values.

➢ The assessment made whether the required assumptions for the application of multiple logistic regression was fulfilled showed that this parsimonious model adequately fits the data as P = 0.317 (by using Hosmer and Lemeshow test).

**Table 4:** Association between knowledge of mothers on PMTCT of HIV and each explanatory variable (Crude & adjusted OR), Gondar, North West Ethiopia, 2011

**Explanatory Variable**	**Knowledge on PMTCT of HIV**		**Crude OR (95% CI)**	**Adjusted OR (95% CI)**	**P-value**
	Yes(1)	No(0)			
**Age**					0.012
15-24	158	20	7.110(3.231,15.644)	3.885 (1.511, 9.991)	
25-34	156	28	5.014 (2.361,10.649)	3.773 (1.397, 10.192)	
35-49	20	18	1.00	1.00	
**Residence**					
Urban	266	19	9.676 (5.333,17.556)	3.620 (1.726, 7.593)	0.001
Rural	68	47	1.00	1.00	
**Education level**					0.009
No Education	138	56	1.00	1.00	
Primary education	31	7	1.797 (0.748, 4.320)	0.683 (0.241, 1.938)	
Secondary & above	165	3	22.319(6.836,72.874)	6.152(1.747, 21.658)	
**Perceived risk of HIV**					0.006
Yes	230	31	2.497 (1.461, 4.268)	2.614 (1.321, 5.170)	
No	104	35	1.00	1.00	
**Have Comprehensive Knowledge on HIV/AIDS**					
Yes	221	18	5.215 (2.899, 9.382)	2.859 (1.405, 5.815)	0.004
No	113	48	1.00	1.00	
**Accessibility of Health facility**					
Yes	277	30	5.832 (3.324, 10.231)	2.163 (1.025, 4.567)	0.043
No	57	36	1.00	1.00	

➢ For explanatory variables having more than two categories, the overall significance is given by their corresponding P-values.

➢ The assessment made whether the required assumptions for the application of multiple logistic regression was fulfilled showed that this parsimonious model adequately fits the data as P = 0.287 (by using Hosmer and Lemeshow test).

Compared to older women (35-49 years), women aged 15-24 years were 4.48 times

[OR & (95%CI) = 4.48(1.45, 13.79)] more likely to have better knowledge on MTCT of HIV in Gondar town, North West Ethiopia.

Compared to women who live in the rural areas, those women living in the urban areas were about 2.46 times [OR & (95%CI) = 2.46 (1.19, 5.09)] more likely to have better knowledge on MTCT of HIV in Gondar town, North West Ethiopia.

Women with education level of secondary and above were 6.85 times [OR & (95%CI) = 6.85 (1.96, 24.01)] more likely to have better knowledge on MTCT of HIV than those with no education.

Women who attend antenatal care service in hospitals were 4.49 times [OR & (95%CI) = 4.49 (1.003, 20.061)] more likely to have better knowledge on MTCT of HIV than those who attended antenatal care in health centers.

Compared to women who do not have comprehensive knowledge on HIV, women with good comprehensive knowledge of HIV were 2.86 times [OR & (95%CI) = 2.86 (1.41, 5.82)] more likely to have better knowledge on PMTCT of HIV.

Women who have perceived risk of HIV were 2.61times [OR & (95%CI) = 2.61 (1.32, 5.17)] more likely to have better knowledge on PMTCT of HIV than those who do not have.

Finally those women who can access health facility (with in 5 km of distance) were about 2.16 times [OR & (95%CI) = 2.16 (1.03, 4.57)] more likely to have better knowledge on PMTCT of HIV than those who do not have accessible health facility.

## Discussion

This study assessed the knowledge of pregnant mothers on MTCT and PMTCT of HIV/AIDS in Gondar town Health facilities, North West Ethiopia. Three hundred fifty four (88.5%) mothers in this study know that HIV can be transmitted from an infected mother to her baby. This finding is also consistent with a finding in Addis Ababa where 89.8% of mothers knew MTCT of HIV but higher than the findings in China, Ghana and six regions of Ethiopia where 57%, 25% and 38.8% knew MTCT of HIV respectively [7-11].

Most of the mothers (83.5%) knew that MTCT of HIV could be prevented by use of ARV drugs (58.4%), by abstaining from breast feeding (18%) and by cesarean section delivery (11%) and this finding is higher than the findings from Addis Ababa and Arba-Minch where 76.8% in Addis Ababa knew MTCT of HIV is preventable and only 20% knew the protective effect of ARV drugs in Arba-Minch [11,12]. This might also be the result of improvements in awareness of MTCT and PMTCT over time.

Positive association was reported between having knowledge on PMTCT of HIV and perceived risk of HIV and comprehensive knowledge on HIV on the other hand. One possible interpretation for this positive association is that those women who do not have comprehensive knowledge on HIV and perceived risk of HIV may fail to appreciate the prevention strategies of mother to child transmission of HIV or may have less access to PMTCT services as well as to health education and promotion in general.

This study has shown a negative association between maternal age and knowledge on MTCT and PMTCT of HIV and this could be that younger and older women may differ in their perceived risk of HIV and understanding of the importance of prevention methods for MTCT of HIV.

Accessibility of health facility (with in 5 km distance) was positively associated with having better knowledge on PMTCT of HIV. The possible explanation for this association could be that the less a health facility is far away from the woman’s house the more a pregnant woman comes in contact with the health center and the more likely she is to hear about PMTCT, among other preventive messages and services. Improving access to and consistent use of ANC is therefore a high priority for improving PMTCT of HIV as shown in Thailand [13].

Women attending ANC in hospitals were also found to have better knowledge than those who attend at health centers and this could be that health education and promotion in MTCT and PMTCT of HIV given in hospitals might be better than health centers or might be since hospitals are found in urban areas those who come to hospitals are more of from urban areas and hence these group of women are different from those who are in the rural area in their socio-economic and educational status. This explanation is in line with the finding in this study that those women are residing in urban areas were more likely to have better knowledge on MTCT and PMTCT of HIV than those who are in the rural area.

Regarding association of education level with MTCT and PMTCT of HIV in this study, those women with secondary and above education level were more likely to have better knowledge on MTCT and PMTCT of HIV than those with no education and this might be due to the fact that uneducated women might be different from educated once in their understanding of MTCT and PMTCT. This possible explanation is also in line with the finding from China and Addis Ababa, Ethiopia where women having secondary and above education level were found to have better knowledge on MTCT and PMTCT of HIV than those with no education [7,11].

### Limitations

Being a cross-sectional survey, causality cannot be inferred from these findings. The study is limited by being facility based and therefore precludes generalization to all pregnant women in Ethiopia indicating a need for further study of knowledge on MTCT and PMTCT of HIV using a more representative sample of pregnant women in the country. Despite this limitation, the study provides useful information that will inform health service planners to design a strategy to increase the awareness of pregnant mothers on MTCT and PMTCT of HIV in Ethiopia.

## Conclusion

Most of the study participants in this study knew that HIV could be transmitted from an infected mother to her baby. Majority of them knew that it is possible to prevent mother to child transmission of HIV and most said that it is by giving prophylactic anti-retroviral drugs. But still a considerable percentage of mothers do not have knowledge of MTCT and PMTCT. This indicates, therefore, the need for exerting more effort to teach mothers about MTCT & PMTCT of HIV and there should be well functioning and accessible health facilities in the country especially in the rural areas.

## Competing interests

The authors declare that they have no competing interests.

## Authors' contributions

MT was investigator, involved in proposal writing, designing, and recruitment and training of supervisors and data collectors, analysis and write-up and in all stages of the project implementation. He did most of the analysis and write up of the paper. GD contributed in the designing of the methodology, lead investigator and involved in designing of project proposal, design of questionnaires, supervision and involved in the analysis stage of the project and final approval of the paper. Both authors read and approved the final manuscript.

## Pre-publication history

The pre-publication history for this paper can be accessed here:

http://www.biomedcentral.com/1471-2393/12/73/prepub
